# FQC Dashboard: integrates FastQC results into a web-based, interactive, and extensible FASTQ quality control tool

**DOI:** 10.1093/bioinformatics/btx373

**Published:** 2017-06-09

**Authors:** Joseph Brown, Meg Pirrung, Lee Ann McCue

**Affiliations:** 1 Earth and Biological Sciences Division, Pacific Northwest National Laboratory, Richland, WA, USA and; 2 Computation and Analytics Division, Pacific Northwest National Laboratory, Richland, WA, USA

## Abstract

**Summary:**

FQC is software that facilitates quality control of FASTQ files by carrying out a QC protocol using FastQC, parsing results, and aggregating quality metrics into an interactive dashboard designed to richly summarize individual sequencing runs. The dashboard groups samples in dropdowns for navigation among the data sets, utilizes human-readable configuration files to manipulate the pages and tabs, and is extensible with CSV data.

**Availability and implementation:**

FQC is implemented in Python 3 and Javascript, and is maintained under an MIT license. Documentation and source code is available at: https://github.com/pnnl/fqc.

## 1 Introduction

Quality control (QC) tools geared towards next-generation sequencing need to adapt to an ever-changing list of requirements and the expectations of different users. Many tools have been developed that address specific QC requirements, though few provide comprehensive results. We address some of these shortfalls through data aggregation and visualization within an extensible and interactive dashboard that simplifies the identification of points of failure for the individuals generating data as well as providing data quality metrics to the researchers using the data for downstream analyses.

FASTQ files containing nucleotide sequence data with associated quality scores can be summarized in several ways. FastQC (http://www.bioinformatics.babraham.ac.uk/projects/fastqc/) is a widely-adopted application in FASTQ QC as it summarizes read quality by position, informs the user of adapter content in sequences, reports on tetramer frequencies, and many other aspects one would expect to glean from the raw sequence data.

FQC Dashboard addresses QC capturing by utilizing FastQC on the backend, taking advantage of its speed and versatility in analyzing single files, and placing those results within a web server with extensible capabilities. FQC’s web framework is built around the same standard metrics obtained from FastQC, but with the ability to aggregate results of multiple sequence runs and easily navigate between them. FQC is intended to be used as an interactive website with dynamically generated plots, which can be readily extended to include additional sequence runs and to add custom plots. This extensibility, and the ability to aggregate multiple sequence runs as individual pages, distinguishes FQC from similar tools (e.g. MultiQC; Ewels *et al.*, 2016) designed to summarize FASTQ data sets as individual or groups of samples, but that lack the ability to display multiple, single-sample reports in a unified dashboard; the goal of this design is to enable sequencing facility personnel to identify and track issues with runs. 

## 2 Approach

FQC Dashboard is a combination of a command line interface (CLI) written in Python 3, which depends on FastQC for processing FASTQ files, a frontend website written in JavaScript, HTML, and CSS, that utilizes Highcharts (http://www.highcharts.com) and D3 (https://d3js.org) Javascript libraries for plotting, and Bootstrap.js (https://getbootstrap.com) for styling and interactivity. These packages are included in the source code repository. The CLI is a single executable with submodules for (i) FASTQ processing of either single- or paired-end data, (ii) batch FASTQ processing based on directory searching and (iii) adding custom plots onto existing dashboard pages. The only dependencies outside of cloning the source code repository are Python 3, which can be installed using Anaconda (https://www.continuum.io/downloads), and FastQC, which can be installed using the Bioconda channel (https://github.com/bioconda/bioconda-recipes).

Plots and tables are dynamically generated from CSV files and configured by the user to be displayed within FQC's supported visualizations: table, line plot, bar plot, area range plot, heatmap or plate heatmap. Configuration is set using JSON files which define the biological samples and sample groups as well as which plots to display and their respective user settings. The default settings for FQC will generate a standard QC analysis from FastQC without the need for the user to edit files, though by modifying the configuration files, the user can personalize and add plots relevant to their analysis protocol.

## 3 Application

An FQC analysis starts with the CLI, which simplifies executing QC on FASTQ files by aggregating QC data like quality by position, sequence content, GC content, and adapter content, and generating the FQC dashboard's configuration files. The default configuration files and settings are determined by the results of FastQC per sample and written to the user’s server data directory. The CLI handles combining paired-end QC data by compiling data from the forward and reverse strand into single plots where applicable, or adding R1 and R2 tabs for more complex plots that should not be combined, like sequence content.

**Fig. 1:**
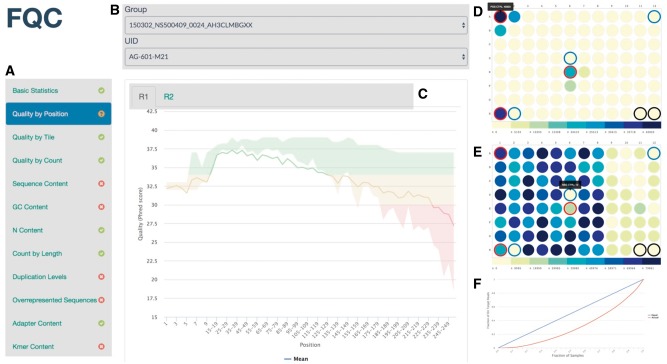
The FQC dashboard. **(A)** Tab navigation with the metric name displayed on the tab and status of the metric as determined by FastQC. **(B)** Dropdown menus of the groups (in this example, a group represents a flow cell) and biological samples within a group available for viewing. **(C)** Plot tabs enable switching between multiple plots (R1 and R2 reads in this example) within a given quality metric. **(D)** 96-well reagent plate heatmap with positive controls encircled in red; this example shows positive control sample bleed-over into neighboring plate wells. **(E)** Read abundance values across a reagent plate showing potential dispensing robot failure after column 8. **(F)** A Lorenz curve showing the distribution of on-target reads across sequence barcodes. A live FQC example site is available at: https://pnnl.github.io/fqc/

The dashboard (Fig. 1) provides a single place for laboratory personnel and researchers to explore the data generated from the command line. Dashboard viewing selections are made from groups (Fig. 1B), which are sets of samples, and then from a list of the samples, which can be comprised of single-end or paired-end sequence QC. Samples are selected and explored individually (Fig. 1B) and can contain many plot pages, each of which can have many subplots which are displayed as tabs in the plot window (Fig. 1C). The user has full control over the parameters for every plot, including axes labels, legend colors and labels, plot color zones, titles, subtitles and tab names.

The extensibility of the dashboard provides various ways to employ the visualizations in practice. In a sequencing facility, FQC can be used to plot abundances across reagent plates to show the spatial relationships of read abundance and illustrate effects of a positive control (Fig. 1D) or view amplification bias per plate (Fig. 1E), crucial information for internal QC. The distribution of reads across barcodes can be viewed as a Lorenz curve to quickly evaluate loading concentrations (Fig. 1F). Additional associated data or summary data that is not suitable for plotting can be displayed as a table with pagination and sortable columns.

## 4 Conclusion

FQC can track standard FASTQ quality metrics while serving a website and being trivially extensible with additional CSV data. The CLI wraps FastQC and builds the website with default QC metrics upon which one can expand without additional programming. The CLI and dashboard lower the threshold of performing and following up on quality issues that may be apparent upon visual inspection and it promotes evidence-based protocol changes in sequencing facilities to generate better quality data.

## Funding

This research was supported by the Microbiomes in Transition Initiative LDRD Program at the Pacific Northwest National Laboratory, a multi-program national laboratory operated by Battelle for the DOE under Contract DE-AC06-76RL01830.

*Conflict of Interest*: none declared.
